# Neck Circumference Cut-Off Points for Identifying Adiposity: Association with Chronic Metabolic Diseases in Older People

**DOI:** 10.3390/jpm14070710

**Published:** 2024-07-01

**Authors:** Dominique A. Díaz, Lydia Lera, Carlos Márquez, Andrea Valenzuela, Rodrigo Saguez, Gerardo Weisstaub, Cecilia Albala

**Affiliations:** 1 Faculty of Health Sciences, Autonomous University of Chile, Santiago 7500000, Chile; dominique.diaz@uautonoma.cl; 2 Latin Division, Keiser University, Online Education, Fort Lauderdale, FL 33309, USA; 3 Institute of Nutrition and Food Technology (INTA), University of Chile, Santiago 7830490, Chile; rodrigo.saguez@inta.uchile.cl (R.S.); gweiss@inta.uchile.cl (G.W.); calbala@uchile.cl (C.A.); 4 Department of Internal Medicine, Universidad de La Frontera, Temuco 4781176, Chile; 5 Nutrition and Dietetics Degree, Faculty of Medicine, University of Desarrollo, Santiago 7610658, Chile; apvalenzuela@udd.cl

**Keywords:** neck circumference, obesity, abdominal obesity, adiposity, chronic diseases

## Abstract

Background: The leading cause of death in older people is cardiovascular diseases. Several studies have found that neck circumference (NC) is a simple anthropometric marker associated with adiposity. The aim of this study is to estimate and validate NC cut-off points as adiposity markers and analyze their association with cardiovascular and chronic metabolic diseases in older people. Methods: A cross-sectional study in 358 non-disabled, community-dwelling older people (71.7 ± 3.9 years) living in Santiago de Chile and participating in the HTSMayor study was conducted. Measurements of body composition and cardiovascular risks were evaluated. Receiver operating characteristic (ROC) curves and multiple logistic regression models were used to evaluate the association of NC with cardiovascular and chronic metabolic diseases. NC cut-off points were obtained to predict obesity, abdominal obesity, and adiposity. Results: The best performance values of neck circumference relative to obesity and adiposity were obtained with respect to abdominal obesity (40.6 cm in men and 34.2 cm in women). Higher NC values were associated with a higher area under the curve (AUC) for men and women (men: AUC = 0.84; women: AUC = 0.86). NC was significantly associated with a higher risk for diabetes mellitus (OR = 1.95), hypertension (OR = 2.42), acute myocardial infarction (OR = 4.36), and comorbidities (OR = 2.01), and a lower risk for sarcopenia (OR = 0.35). Conclusions: This study shows that NC is a useful tool for detecting abdominal obesity, obesity, and adiposity in older people and that a higher NC increases the risk of chronic diseases.

## 1. Introduction

The accelerated growth of the population aged 65 years and older and the increase in the prevalence of obesity have been accompanied by an increase in chronic diseases. Some of the more prevalent diseases in older people are high blood pressure, hyperlipidemia, and diabetes mellitus, all associated with obesity.

The prevalence of obesity and abdominal obesity has significantly increased worldwide, which constitutes a public health problem due to its direct effects on health and quality of life [1,2]. The early detection of obesity can prevent the impact of its adverse results, such as cardiovascular diseases (CVDs) [3,4]. Cardiovascular diseases are the main cause of death among older people in Chile and globally, with more than 29019 deaths per year, which represents about one-third of all deaths [5].

To diagnose abdominal obesity, experts recommend using waist circumference (WC) and cut-off points for each population or country [6]. The United States criteria (NCEP-ATP III) of a circumference of ≥102 cm in men and ≥88 cm in women are used in Chile, but the cut-off points have not been validated in our population [7]. The Latin American Diabetes Association defines abdominal obesity as a WC of ≥94 cm and ≥90 cm in men and women, respectively [8]. Due to the lack of local information and the harmonization of metabolic syndrome criteria for South American countries, the WHO recommends the parameters of Southeast Asia (≥90 cm in men and ≥80 cm in women) [9,10]. 

Evaluating anthropometric parameters as indicators of nutritional status can be challenging for older adults in primary health care, due to the difficulty of older people undressing and the conditions of the rooms in health care centers. Additionally, other procedures like ultrasound, computed tomography, DXA, and magnetic resonance are costly and primarily used for research purposes [11]. The search for criteria for measuring adiposity with easy clinical applicability has led researchers to consider the importance of investigating other anthropometric parameters that could integrate or replace some already established ones [12]. Thus, the measurement of neck circumference (NC) was investigated due to some limitations that the measurement of the WC presents, such as the lack of uniformity in measurement techniques, variations in certain health conditions, postprandial abdominal distention, or respiratory movements [13]. The neck under normal conditions does not suffer measurement fluctuations throughout the day. It is an easily measurable parameter, and it has some advantages, such as the fact that it does not require undressing the patient or moving the patient; thus, the exam could be performed in less time and preserve the patient’s privacy [14,15,16].

NC has been suggested as a measure for identifying overweight and obesity among children, adolescents, and adults [17,18]. However, there are few studies on the use of NC as an anthropometric risk parameter for cardiovascular or chronic metabolic diseases in older adults [19,20,21,22,23,24,25,26,27,28,29,30,31,32]. In Chile, this measure has not been studied as an anthropometric parameter of adiposity risk in older people. Therefore, the objective of this research is to establish and validate NC cut-off points for adiposity—adiposity via dual-energy X-ray absorptiometry (DXA) and abdominal obesity and obesity using a body mass index (BMI) of ≥30 kg/m^2^—and analyze their association with cardiovascular and chronic metabolic diseases and sarcopenia in a sample of older community-dwelling Chilean adults. 

## 2. Materials and Methods

### 2.1. Design and Participants

A cross-sectional study of 358 community-dwelling people who are 60 years and older (mean ± SD: 71.7 ± 3.9; 73.5% females) living in Santiago de Chile was carried out, with initial measurements of body compositions via a DXA scan (Lunar PRODIGY IDEXA 13,6, GE Healthcare, Chicago, USA) of the HTSMayor study designed to study sarcopenia in elderly Chilean individuals [33]. 

The data were evaluated between 8 August 2016 and 29 June 2017, at the Institute of Nutrition and Food Technology (INTA), University of Chile. Participants were selected from a sample of 430 older adults from the HTSMayor study who had DXA measurements and complete anthropometry. Seventy-two people were excluded from the study due to the presence of a disease or condition that could affect the measurement of NC (e.g., goiter). 

### 2.2. Data Collection

All subjects underwent face-to-face interviews, which included sociodemographic information, the Mini Nutritional Assessment Short-Form (MNA-SF), anthropometric measurements, body composition information, and information on self-reported chronic diseases, among others.

### 2.3. Anthropometric Measurements

Anthropometric measurements, such as weight, height, knee height, calf circumference (CC), WC, hip circumference (HC), handgrip strength, and NC, were evaluated according to the methods described in a previous study [34]. Handgrip strength was measured via handgrip dynamometry (JAMAR dynamometer), and the best of two measurements was recorded with the dominant hand [35]. NC was measured on the cricoid cartilage, with the tape perpendicular to the longitudinal axis of the neck [36].

### 2.4. Obesity, Abdominal Obesity, and Adiposity

Obesity was calculated according to the body mass index (BMI, kg/m^2^). Overweight was considered when the BMI was ≥25 and <30 kg/m^2^, and obese was considered when the value was ≥30 kg/m^2^ [37]; abdominal obesity was considered according to ATPIII (WC > 102 cm in men; >88 cm in women). Adiposity was determined by applying dual-energy X-ray absorptiometry (DXA) using a Lunar PRODIGY densitometer (p60 fat mass ≥25 kg in men and ≥30 kg in women).

### 2.5. Chronic Diseases

Self-reported pathologies of high prevalence and those associated with high cardiovascular risks were selected, such as type 2 diabetes mellitus (DM2), arterial hypertension (HT), and cardiovascular and chronic metabolic diseases. Cardiovascular disease classification was based on previous clinical diagnoses of acute myocardial infarction (AMI) and cerebrovascular accident (CVA). Blood pressure values were measured, recorded, and classified as follows: elevated blood pressure was considered ≥140/90 mmHg [23]. Multimorbidity was defined as having two or more self-reported chronic diseases (defined as two or more self-reported diseases: high blood pressure, diabetes, coronary heart disease, stroke, chronic obstructive pulmonary disease, cancer, and arthritis). 

### 2.6. Symptoms of Depression

Symptoms of depression were determined using the 15-item Geriatric Depression Scale (GDS-15).

### 2.7. Sarcopenia

The diagnosis of sarcopenia was carried out using HTSMayor software 1.0 [27], a version adapted from the diagnostic algorithm of sarcopenia proposed by the European Working Group on Sarcopenia in Older People (EWGSOP1) in 2019 [38]; it considers low physical levels, low-force muscles, and/or low muscle mass. Low physical performance was determined via the three-meter walking speed test. In this test, the subject walked three meters at a normal pace, with technical aids if required; the pace was measured from the time that they started walking, and velocity was calculated as the distance divided by time, with a cut-off of 0.8 m/sec. Muscle strength was measured according to handgrip dynamometry with Chilean cut-off points (men: <27 kg; women: <15 kg). Low muscle mass was estimated with the cut-off points of the skeletal mass index (SMI) obtained for the Chilean population through DXA measurements (men: <7.19 kg/m^2^; women: <5.77 kg/m^2^). 

### 2.8. Mini Nutritional Assessment Short-Form (MNA-SF)

MNA-SF was used to measure malnutrition or the risk of malnutrition, an instrument validated in Chile. Subjects were classified as normal or well-nourished if the score was ≥12 (12–14) points, at nutritional risk if the score was 8–11 points, and malnourished if the score was ≤7 points [39]. 

### 2.9. Statistical Analysis

Continuous variables were expressed as the mean ± standard deviation (SD), and categorical variables were absolute and relative frequencies expressed as percentages. The difference between sexes was calculated using a *t*-test for two independent samples or Pearson’s chi-square test. Pearson or Spearman correlation tests were performed to analyze the association between NC with respect to age and body composition measures (BMI, WC, and DXA). Receiver operating characteristic (ROC) curves were used to calculate NC cut-off points in relation to obesity according to the WHO (≥30 kg/m^2^), abdominal obesity according to ATPIII, and adiposity according to DXA (p60 fat mass). Multiple logistic regression models were performed to determine the risk of elevated NC values with cardiovascular and chronic metabolic diseases, adjusted for age, sex, tobacco consumption, lean mass–fat ratio, nutritional status, and MNA-SF. The Hosmer–Lemeshow test was used to assess the goodness of fit for the estimated models. All statistical analyses were performed with STATA 15.0 (StataCorp.2017. Stata Statistical Software: Release 15. College Station, TX: StataCorp LP). 

### 2.10. Ethical Considerations

The study and informed consent form were approved by the Ethics Committee of the Institute of Nutrition and Food Technology (INTA), University of Chile with the ethical approval code act number 4 dated 16 March 2016. Before any procedures were performed, all subjects signed a consent form.

## 3. Results

### 3.1. Sample Description

Table 1 presents the sociodemographic and health characteristics of the sample according to sex. The sample included 358 people over 60 years old with a mean age of 71.7 ± 3.9 y, which was similar in both sexes, and it had a higher percentage of women (75.3%). The mean years of education was also similar in men and women. Cardiovascular complications were higher in men than women (AMI: 21.1% vs. 11.0%; CVA 7.3% vs. 3.4%), and symptoms of depression and osteoarthritis were higher in women than in men. Moreover, the prevalence of multiple chronic diseases was higher in women than men (61% vs. 76%). Statistical differences were not found between men and women with respect to physical activity, smoking, hypertension, diabetes, and sarcopenia.

### 3.2. Body Composition

As observed in Table 2, BMI was similar in men and women (29.7 kg/m^2^ vs. 30.0 kg/m^2^). However, 81.9% of the studied population presented malnutrition due to excess values (38.3% overweight and 43.5% obese). When comparing the nutritional evaluation with respect to MNA-SF, it is observed that 72.3% were classified with a normal nutritional status. In addition, men had higher handgrip strength than women, together with a higher value of trunk and total lean mass, WC, WC/HC ratio, NC, and CC. 

Additionally, NC presented a strong positive correlation with body composition measures in both sexes: BMI: men = 0.72 and women = 0.7, with a total of 0.56; WC: men = 0.73 and women = 0.65, with a total of 0.63; and Pearson’s correlation coefficient at *p* < 0.01.

### 3.3. Cut-Off Points for Neck Circumference

**Figure 1:**
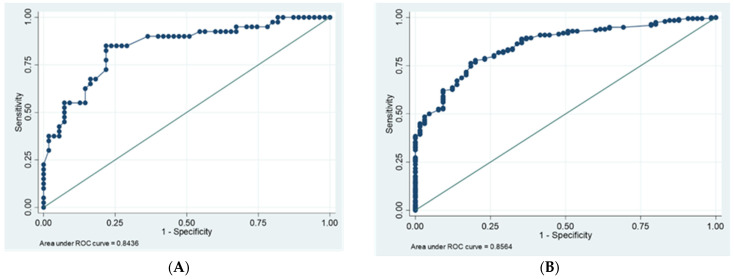
ROC curves for the prediction of obesity risk by neck circumference according to waist circumference (men > 102 cm and women > 88 cm). (**A**) men and (**B**) women. AUC, area under the curve; ROC, receiver operating characteristics.

Table 3 describes the optimal NC cut-off points obtained via ROC analysis using cut-off values that predict abdominal obesity according to WC (men > 102 cm and women > 88 cm), obesity according to BMI (≥30 kg/m^2^), and adiposity measured according to DXA (60th percentile for fat mass: men ≥ 25 kg and women ≥ 30 kg), with high sensitivities and specificities. The area under the curve (AUC) of the NC concerning abdominal obesity was higher than 0.8 in both sexes (men = 0.84 and women = 0.86), and the highest value was observed when compared to the NC associated with obesity and adiposity. The optimal cut-off values for men correspond to 40.6 cm (CI:0.76–0.92; *p* < 0.001; sensitivity: 0.85; specificity: 0.78), with 34.2 cm for women (CI:0.81–0.90; *p* < 0.001; sensitivity and specificity: 0.78). When analyzing the neck with respect to adiposity and obesity, the optimal cut-off values for men correspond to 41.4 cm and 40.5 cm and for women 36.9 cm and 35.5 cm. The ROC curves for the prediction of obesity risk are shown in Figure 1.

### 3.4. Crude Associations between Diseases and Neck Circumference Cut-Off Points 

Table 4 shows the crude associations between NC cut-off points for abdominal obesity, obesity, and adiposity and cardiovascular and chronic metabolic diseases according to sex. The crude analyses carried out found significant associations between women with higher NC values and DM2 and HT for abdominal obesity, obesity, and adiposity. Women with higher NC values had AMI with abdominal obesity (OR = 8.4) and osteoarthritis (OR = 1.79). Lower NC values increased the risk of presenting sarcopenia with abdominal obesity (OR = 0.28), obesity (OR = 0.28), and adiposity (OR = 0.29).

### 3.5. Associations between Diseases and Adjusted Neck Cut-Off Points

Multivariate logistic regression analysis showed that higher NC values for abdominal obesity were associated with AMI, DM2, HTA, and multimorbidity. The models were adjusted according to sex, age, lean mass–fat ratio, nutritional status (BMI and MNA-SF), and tobacco consumption. In the adjusted model, higher NC values are a risk factor for AMI (OR = 4.36), DM2 (OR = 1.95), and multimorbidity (OR = 2.01) and are not risk factors for sarcopenia (OR = 0.35) (Table 5). Multivariate logistic regression analysis was also used to evaluate the associations between optimal NC values and sarcopenia.

## 4. Discussion

### 4.1. Principal Findings

In this study, NC cut-off points were estimated and validated as indicators of obesity, abdominal obesity, and adiposity, which were measured via DXA and by means of ROC curves in older people. Associations between NC and cardiovascular and chronic metabolic diseases were found. NC was associated with weight, BMI, waist and hip circumferences, total lean mass, trunk fat mass, and total fat mass in both sexes. Only men presented positive associations between NC and calf circumference and lean trunk mass. The cut-off points calculated for NC with respect to abdominal obesity, obesity, and adiposity were similar (men: 40.6 cm and women: 34.2 cm; men: 40.5 cm and women: 35.5 cm; men: 41.4 cm and women: 36.9 cm, respectively). The area under the curve (AUC) of NC associated with abdominal obesity showed the highest values in both sexes (men: AUC = 0.84; women: AUC = 0.86). The optimal NC cut-off value for predicting obesity or adiposity was observed with abdominal obesity. The crude and adjusted analysis of the factors associated with increased NC exhibited an association with a higher risk of presenting DM2, HT, and comorbidities in women, calculated for abdominal obesity, obesity, and adiposity, than in men.

Values similar to NC were obtained in Brazil by Coelho et al. They studied 435 people over 60 years of age and calculated the cut-off points according to BMI (men = 40.5 cm; women = 35.7 cm), and women with high NC values were associated with high levels of mean arterial pressure and type 2 diabetes mellitus [20]. 

The cut-off points related to abdominal obesity were protective against sarcopenia. Thus, they were estimated as cut-off points for a lower NC (men: 39.4 cm; women: 33.0 cm), suggesting that these cut-off values point to an increased risk of having sarcopenia after adjusting for several variables. More studies should be carried out, since Machino et al. (2021) found that NC was significantly associated with presarcopenia [40]. 

In the 2016–2017 National Health Survey (NHS) in Chile supplemented with an anthropometric module with the measurement of the neck circumference, this measure was associated with a better estimate of the prevalence of obstructive sleep apnea syndrome (OSAS) [41]. Similar results were obtained by Cielo et al. (2020), who observed that neck fat is associated with obesity and neck circumference in adolescents and is greater in females versus males [42]. Caro et al. (2019) used the data from the NHS and estimated NC cut-off points associated with cardiovascular risk in the Chilean population with a mean age of 47.6 years [43]. Another study carried out in Brazil by Nogueira showed that NC was positively correlated with WC, BMI, waist–hip ratio, the percentage of total body fat, and insulin resistance (IR) in both sexes. In the case of women, NC showed the highest AUC for insulin resistance, and in men, WC showed the highest AUC, followed by BMI. NC values of ≥39.4 cm for men and ≥33.7 cm for women were the best cut-off values for identifying subjects with IR; the authors suggest the use of NC as a predictor of IR in older adults [25].

He et al. (2022) studied the association between NC, BMI, WC, and T2DM. They results demonstrated that NC is closely related to BMI, WC, and components in T2DM. The cutoff points of NC can identify all components in males and hyperuricemia in females with the same efficiency as WC [26]. Yang et al. (2019) found, in a large sample of older people (n = 2646), that an increased NC value is a risk factor for developing type 2 diabetes in elderly Chinese individuals [27]. Data from Moura et al., who evaluated 15085 Brazilians from the Brazilian Longitudinal Study of Adult Health (ELSA-Brasil) baseline data, estimated sex- and age-specific quantile values for NC and WC according to BMI. There was significant dispersion in WC and NC values for a given BMI and age strata for both men and women [24]. Likewise, Baena et al. (2016), who studied a sample of 8726 Brazilian adults, found that men and women with large N values (≥40 cm and ≥34.1 cm, respectively) were more likely to have insulin resistance, low HDL cholesterol levels, elevated blood pressure, and high triglyceride levels [18]. Recently, Mendes et al. (2021) studied the relationship between waist and NC, with several metabolic parameters showing that both are reliable tools for diagnosing metabolic syndrome (MetS) in Brazilian patients [28].

Zhang Y et al. (2020) established that the neck circumference was positively correlated with waist circumference, BMI, fasting blood glucose, triacylglycerol, and LDL-Cholesterol [17]. Fu et al. (2019) found that NC was significantly associated with cardiometabolic disease in 4000 Chinese participants with a mean age of 56.0 ± 9.8 years [29].

Studies such as Yang’s or Tibana’s agree with data from Koppad et al. (2017), who studied the metabolic risk estimated by the Framingham risk score and the risk of Coronary Artery Disease (CAD) in subjects based on NC; their results shows NC gives a simple and easy prediction of CAD risk and is more reliable than traditional risk markers like BMI [27,30,31]. Namazi et al. (2018) carried out a systematic review and meta-analysis of the association between NC and MetS and its components in adult populations. They found that people with higher NC values had two times the risk of hypertriglyceridemia compared to those with lower NC positive associations with respect to concentrations of BMI, WC, and HTA as well as other lipid profiles, such as SBP, DBP, and FBS. An inverse association between NC and serum HDL-C levels was also observed; however, heterogeneity was considerably high. Moreover, they did not find MetS risks in the adult populations included in our review [32]. Although the present study cannot clarify the mechanisms that may explain the association of NC with DM2 and HT due to its design, evidence in the literature allows inferences to be made, and studies have shown positive associations between NC and fasting triglyceride, glucose, insulin, adiponectin, glycosylated hemoglobin, and blood pressure values and the thickness of the carotid intima–media, among others [21,22,23].

Furthermore, NC has been suggested to be a surrogate marker for upper body subcutaneous fat, which is more lipolytically active than lower body fat, due to its association with insulin resistance, glucose disturbances, atherosclerosis, and endothelial dysfunction [44]. Therefore, it is a powerful marker of visceral adipose tissue, since a greater bioavailability of free fatty acids can be suggested as a common pathway in the relationship between NC, DM2, and HTN. The determination of the NC cut-off values is justified, considering abdominal obesity as an indicator (men: 40.6 cm; women: 34.2 cm). Moreover, Tanaka et al. (2020) found that NC can be a marker of frailty in elderly women [45].

The availability of NC cut-off points, especially in this population, suggests that this anthropometric measurement is more appropriate among the elderly than other evaluations since it can be performed with the subject in a sitting or standing position. Light clothing is also not required, it has a shorter realization time, and only one tape measure is required as a measuring instrument; therefore, this evaluation method has lower costs. In addition, this measurement method does not show variability during the day, and it is not affected by abdominal distention after food intake or the breathing phase (inhalation or exhalation). Older people can be evaluated even in conditions that impair their functionality, such as a decrease in the ability to remain in an upright position brough on by frailty, sarcopenia, osteopenia, osteoporosis, arthritis, different types of pain, or weakness of the skeletal muscle. Even at the hospital level, we can find elderly people in critical situations that prevent lifting of the patient. In addition, complications such as chronic obstructive pulmonary disease, edema, or ascites are present, and thus weight measures can be invalidated. Therefore, the NC is a simple tool that is useful for measurements, even in the supine position. 

In this study, we found NC cut-off points and showed that NC is a useful marker of central obesity, obesity, and adiposity in older people. Moreover, increased values of NC are associated with a higher risk for chronic diseases. Additionally, we observed that sarcopenia and obesity are independent in males but negatively correlated in women; the reason may be explained by gender differences, particularly in body composition.

### 4.2. Strengths and Limitations

The first limitation of the present study is that it is a cross-sectional study; thus, no causal associations were established. The second limitation is the use of self-reported data to quantify the prevalence of chronic diseases. The use of biochemical measurements could have resulted in a better understanding of the association between NC and cardiovascular risk factors and chronic diseases in the elderly. Another limitation is that we only had the diagnosis report of cardiovascular pathologies such as high blood pressure and type 2 diabetes mellitus; we did not have the values of biomarkers such as glycemia to establish a cut-off point linked to such parameters. Thus, it is necessary to carry out further research that could relate these parameters to an evaluation of metabolic control.

Some strengths of this study are that it is the first to determine the cut-off points of neck circumferences in older people, and it reports the associations between the cut-off points and cardiovascular and chronic diseases in Chile. Due to these observations, the neck circumference is an alternative and innovative anthropometric measurement factor that saves time. It is non-invasive, inexpensive, and reliable, making it an easy tool for use in clinical practice as a predictor of adiposity and cardiovascular risk. We found that higher NC values for abdominal obesity (≥40.6 cm in men and ≥34.2 cm in women) are significantly associated with a greater risk of comorbidities, such as DM2, HT, and AMI, and a lower risk of sarcopenia.

## 5. Conclusions

In conclusion, we estimated and validated NC cut-off points as a useful screening tool to detect abdominal obesity and sarcopenia in older people. These values can be used by health staff as part of a preventive medical exam for older adults in public and private healthcare centers, because increased NC values are associated with chronic and cardiovascular diseases, such as DM2, HT, and AMI. Women with increased NC values present a higher risk of DM2, HT, AMI, and osteoarthritis than men. Moreover, in women, lower NC values are associated with sarcopenia risk.

## Figures and Tables

**Table 1:** Sociodemographic and health characteristics by sex.

Variables	Men *n* = 95	Women *n* = 263	Total *n* = 358	*p*-Value ^1^
Age (years), mean ± SD	71.8 ± 4.3	71.65 ± 3.7	71.7 ± 3.9	0.6
Education (years), mean ± SD	9.1 ± 4.7	8.9 ± 4.5	8.97 ± 4.5	0.6
Physical activity, *n* (%)	73 (82.9)	219 (87.2%)	292 (86.1%)	0.3
Smoking, *n* (%)	15 (15.79%)	34 (12.9%)	49 (13.6%)	0.4
Hypertension, n (%)	70 (73.68%)	195 (74.1%)	265 (74.0%)	0.9
DM2, *n* (%)	32 (33.6%)	79 (30.0%)	111 (31.0%)	0.51
AMI, *n* (%)	20 (21.0%)	29 (11.0%)	49 (13.6%)	0.015
CVA, *n* (%)	7 (7.3%)	9 (3.4%)	16 (4.4%)	0.016
Symptoms of depression (GDS > 5), *n* (%)	12 (12.6%)	105 (40.0%)	117 (32.7%)	<0.001
Osteoarthritis, *n* (%)	10 (10.5%)	76 (28.9%)	86 (24.0%)	0.001
Sarcopenia, *n* (%)	13 (13.6%)	36 (13.6%)	49 (13.6%)	0.9
Multimorbidity, *n* (%)	58 (61.0%)	201(76.7%)	259 (72.5%)	0.003

^1^ Based on *t* test, except categorical variables, which were based on Pearson chi-square test. SD: standard deviation; Physical activity (<3 times/week); DM2: type 2 diabetes mellitus; AMI: acute myocardial infarction; CVA: cerebrovascular accident; GDS: Geriatric Depression Scale; multimorbidity: ≥2 chronic diseases.

**Table 2:** Body composition, nutritional status, and handgrip strength by sex.

Variables	Men *n* = 95	Women *n* = 263	Total *n* = 358	*p*-Value ^1^
BMI (kg/m^2^), mean ± SD	29.6 ± 5.0	29.9 ± 5.6	29.9 ± 5.4	0.6
Nutritional state, *n* (%)				
Underweight (BMI < 20)	1 (1.05)	3 (1.1)	4 (1.1)	0.7
Normal (BMI: 20–24.9)	13 (13.6)	48 (18.2)	61(17.0)	
Overweight (BMI: 25–29.9)	38 (40.0)	99 (37.6)	137 (38.2)	
Obese (BMI ≥ 30)	43 (45.2)	113 (42.9)	156 (43.5)	
Nutritional assessment, using the MNA-SF, *n* (%)				
Malnourished (≤7 points)	1(1.0)	9 (3.4)	10 (2.8)	0.1
Nutritional risk (8–11 points)	19 (20.2)	70 (26.6)	89 (24.9)	
Normal (≥12 points)	74 (78.7)	184 (69.9)	258 (72.2)	
Waist circumference (cm), mean ± SD	101.8 ± 12.0	97.7 ± 13.3	98.8 ± 13.1	0.009
Abdominal obesity (≥88/102 cm), *n* (%)	40 (42.1%)	198 (75.2%)	238 (66.4%)	<0.001
Hip circumference (cm), mean ± SD	102.3 ± 9.3	105.4 ± 11.3	104.6 ± 10.8	0.008
Waist/Hip ratio, mean ± SD	0.9 ± 0.06	0.9 ± 0.07	0.9 ± 0.07	<0.001
Neck circumference (cm), mean ± SD	41.0 ± 3.9	35.7 ± 3.26	37.1 ± 4.1	<0.001
Calf circumference (cm), mean ± SD	37.2 ± 3.3	35.4 ± 3.5	35.8 ± 3.5	<0.001
Trunk lean mass (kg), mean ± SD	24.6 ± 3.5	18.2 ± 3.0	19.9 ± 4.2	<0.001
Lean mass (kg), mean ± SD	50.2 ± 6.8	36.1 ± 4.9	39.9 ± 3.9	<0.001
Trunk fat mass (kg), mean ± SD	16.9 ± 6.4	17.0 ± 6.1	17.0 ± 6.2	0.8
Fat mass (kg), mean ± SD	27.2 ± 9.8	30.1 ± 9.5	29.3 ± 9.6	0.010
Lean mass/fat mass, mean ± SD	2.3 ± 1.0	1.3 ± 0.4	1.5 ± 0.7	<0.001
Handgrip strength (kg), mean ± SD	35.0 ± 8.8	21.3 ± 6.4	25.0 ± 9.3	<0.001
Low handgrip strength (≤27/15 kg), *n* (%)	17 (17.8%)	38 (14.5%)	55 (15.4%)	0.4

^1^ Based on *t* test, except categorical variables, which were based on Pearson chi-square test. SD: standard deviation; BMI: body mass index; MNA-SF: Mini Nutritional Assessment Short-Form.

**Table 3:** Area under the curve (AUC), cut-off value, sensitivity, and specificity of NC in detecting abdominal obesity, obesity, and adiposity according to ROC curves.

Variables	Cut-Off Points (cm)	Sensitivity (%)	Specificity (%)	Classification (%)	AUC
Abdominal obesity according by WC (men > 102 cm and women > 88 cm)	≥40.6	85.0	78.1	81.0	0.84
	≥34.2	78.7	76.9	78.3	0.86
Obesity by BMI > 30 kg/m^2^	≥40.5	76.7	73.0	74.7	0.76
	≥35.5	76.9	73.3	74.9	0.84
Adiposity measured by DXA (60p for fat mass) (men ≥ 25 kg and women ≥ 30 kg)	≥41.4	83.3	74.0	75.7	0.82
	≥36.9	86.36	71.37	72.62	0.85

Notes: ROC: receiver operating characteristics; BMI: body mass index; 60p: DXA 60th percentile for fat mass.

**Table 4:** Crude associations between neck circumference cut-off points and cardiovascular and chronic metabolic diseases, by sex.

	Abdominal Obesity		Obesity According to Body Mass Index		Adiposity Measured by DXA	
	Men ≥ 40.6 cm	Women ≥ 34.2 cm	Men ≥ 40.5 cm	Women ≥ 35.5 cm	Men ≥ 41.4 cm	Women ≥ 36.9 cm
**Diseases**	**OR (95% CI)**	**OR (95% CI)**	**OR (95% CI)**	**OR (95% CI)**	**OR (95% CI)**	**OR (95% CI)**
CVA	0.8 (0.20–3.18)	0.66 (0.17–2.53)	0.84 (0.21–3.33)	1.44 (0.38–5.49)	1.57 (0.39–6.3)	1.01 (0.25–4.15)
AMI	1.71 (0.63–4.67)	**8.4 (1.96–36.34)**	1.4 (0.52–3.76)	2.0 (0.91–4.44)	1.74 (0.64–4.75)	1.76 (0.8–3.84)
Osteoarthritis	1.14 (0.48–2.73)	1.33 (0.81–2.21)	1.21 (0.5–2.9)	**1.79 (1.1–2.93)**	1.51 (0.61–3.73)	1.67 (0.99–2.81)
DM2	1.03 (0.44–2.42)	**3.07 (1.63–5.79)**	1.1 (0.47–2.58)	**3.6 (2.06–6.3)**	1.47 (0.61.3.56)	**2.36 (1.36–4.09)**
HTA	1.99 (0.77–5.11)	**2.67 (1.51–4.69)**	1.99 (0.78–5.11)	**3.28 (1.78–6.02)**	1.99 (0.71–5.61)	**2.94 (1.48–5.85)**
Sarcopenia	1	**0.28 (0.14–0.59)**	1	**0.28 (0.22–0.64)**	1	**0.29 (0.11–0.76)**
Multimorbidity	1.51 (0.66–3.46)	**2.43 (1.35–4.36)**	1.40 (0.61–3.22)	**3.56 (1.87–6.79)**	1.77 (0.72–4.35)	**2.76 (1.36–5.63)**

Notes: OR: odds ratio; CI: confidence interval; CVA: cerebrovascular accident; AMI: acute myocardial infarction; DM2: Diabetes mellitus type 2; HTA: Hypertension; multimorbidity: ≥2 chronic diseases; abdominal obesity according waist circumference (men > 102 cm and women > 88 cm); obesity according to mass index body (BMI ≥ 30 kg/m^2^), and adiposity measured by DXA (60th percentile for fat mass). Bold values represent statistically significant results at *p* < 0.05.

**Table 5:** Logistic models of acute myocardial infarction, type 2 diabetes mellitus, hypertension, and comorbidity with neck circumference cut-off points by abdominal obesity, adjusted by sex, age, lean mass/fat mass ratio, nutritional status, MNA-SF, and smoking.

	Model 1 AMI	Model 2 DM2	Model 3 HTA	Model 4 Multimorbidity	Model 5 Sarcopenia
	OR (95% CI)	OR (95% CI)	OR (95% CI)	OR (95% CI)	OR ( 95% CI)
NC Men/Women ≥ 40.6/34.2 cm	**4.36 (1.70–11.18)**	**1.95 (1.06–3.58)**	1.54 (0.83–2.84)	**2.01 (1.08–3.74)**	**0.35 (0.14–0.92)**
Women	**0.31 (0.12–0.76)**	0.89 (0.47–1.68)	0.62 (0.30–1.26)	1.38 (0.67–2.85)	**0.16 (0.05–0.5)**
Age (years)	1.00 (0.92–1.10)	0.93 (0.88–0.1)	0.98 (0.92–1.05)	0.99 (0.93–1.05)	0.99 (0.9–1.1)
Lean mass/fat mass ratio	0.94 (0.42–2.12)	1.15 (0.76–1.76)	0.76 (0.47–1.22)	0.58 (0.31–1.08)	**0.09 (0.03–0.34)**
Nutritional status (kg/m^2^)					
BMI: <20	-	0.46 (0.03–6.51)	3.04 (0.20–45.82)	0.71 (0.04–13.34)	1.0
BMI: 25–29.9	0.49 (0.16–1.52)	0.82 (0.38–1.77)	1.44 (0.70–2.94)	0.79 (0.36–1.73)	**0.15 (0.06–0.36)**
BMI: ≥30	0.47 (0.14–1.62)	1.10 (0.48–2.51)	1.78 (0.77–4.09)	0.65 (0.26–1.65)	**0.04 (0.01–0.14)**
MNA-SF					
Nutritional risk	1.81 (0.88–3.74)	1.60 (0.94–2.71)	1.35 (0.74–2.5)	**3.64 (1.76–7.5)**	1.14 (0.51–2.55)
(8–11 points)	**16.67 (3.30–84.23)**	1.48 (0.33–6.7)	1.38 (0.23–8.2)	6.52 (0.49–87.51)	1.01 (0.11–9.3)
Malnourished	**4.8 (1.04–22.23**)	1.54 (0.73- 3.29)	1.42 (0.69–2.92)	**2.96 (1.35–6.49)**	0.99 (0.33–3.03)

Notes: NC: neck circumference; AMI: acute myocardial infarction; DM2: type 2 diabetes mellitus; HTA hypertension; multimorbidity: ≥2 chronic diseases; OR: odds ratio; CI: confidence interval; BMI: body mass index; MNA-SF: Mini Nutritional Assessment Short-Form. Reference categories: Nutritional status: BMI: 20–24.9 kg/m^2^; MNA-SF: ≥ 11 points. Hosmer–Lemeshow goodness-of-fit test: *p* > 0.5, indicating the goodness of fit of the models are satisfactory. Bold values represent statistically significant results at *p* < 0.05.

## Data Availability

Data supporting reported results can be found upon request to corresponding authors.
